# Spatio-temporal Analysis of Anthropogenic Disturbances on Landscape Pattern of Tourist Destinations: a case study in the Li River Basin, China

**DOI:** 10.1038/s41598-019-55532-w

**Published:** 2019-12-17

**Authors:** Yunyun Xiang, Jijun Meng, Nanshan You, Peixiong Chen, Hui Yang

**Affiliations:** 1 Second Institute of Oceanography, Ministry of Natural Resources, Hangzhou, 310012 China; 2 School of Urban and Environmental Sciences, Key Laboratory for Earth Surface Processes, Ministry of Education, Peking University, Beijing, 100871 China; 3 Institute of Geographic Sciences and Natural Resources Research, CAS, Beijing, 100101 China

**Keywords:** Ecological modelling, Environmental impact

## Abstract

The impact of human-related activities on the eco-environment of tourist destinations is an important part of recreation ecology research. However, traditional studies have mainly concentrated on the static influences upon the simple factors of soil or vegetation in tourist destinations, and the relationship between anthropogenic disturbances and landscape patterns is little understood. In this study, we constructed a disturbance model on a landscape scale to identify and quantify the main anthropogenic disturbances. The overall variation coefficient (*OVC*) index is defined as the intensity of different disturbance sources, and landscape structure analysis methods are used for temporal and spatial differentiation, which is applied in the Li River Basin, China. Three typical types of human-related activities are identified as possible anthropogenic disturbance sources in the region, and their notable influential spheres are determined. Then, the dynamic changes in tourism disturbance in two periods and the spatial distribution characteristics related to three factors are explored. The results suggest that settlement and tourism disturbances have exerted considerable impacts on landscape patterns, and the differentiation characteristics are closely related to local tourism development policies and patterns. The disturbance model could be applied in other tourism destinations and provide countermeasures for regional tourism management.

## Introduction

Disturbance is a ubiquitous event or process in nature, occurring on varied spatial and temporal scales, which can immediately influence ecosystem succession and landscape patterns^1–4^. It can be divided into natural and anthropogenic disturbances based on the source of the disturbance. Natural disturbance, such as climate change, natural disasters, and volcanic eruptions, refers to a natural phenomenon or event without any human interference, while anthropogenic disturbance is the purposive transformation of local natural environments by humans, such as field burning, deforestation, overgrazing, and road and scenic spots construction^5–8^. The identification of disturbance is highly dependent on the scale of the system, because a disturbance in a micro-scale system would just be part of the normal phenomena in a macro-scale system^9^. It has been shown that on a micro scale, i.e. at sites or patches, anthropogenic disturbances could become the crucial driving forces of soil erosion and vegetation degradation^10^. In recent years, due to the universality of and variability in human activities^11^, the intensity of anthropogenic disturbance, which includes resource destruction, ecological fragmentation and landscape degradation, is constantly increasing and exceeds the capacity of landscapes^12,13^. Therefore, recognizing the mode and intensity of anthropogenic disturbances and analysing the corresponding ecosystem fragmentation processes could make a large difference in the maintenance of ecosystem sustainability and integrity.

Apart from the disturbance of local residents’ activities, tourism exploitation, especially scenic spots construction, has become an increasingly complicated form of anthropogenic disturbance in typical tourist destinations, as it requires finite and valuable natural resources^14^, such as biological resources, land structures, air quality, and human and cultural environmental resources^15,16^. In recent decades, the degradation and destruction of landscapes in tourist destinations caused by anthropogenic disturbances has been growing increasingly acute^17^, directly affecting the ecosystem balance and landscape sustainability. For the coordinated development of tourism exploitation and landscape protection, how to quantitatively evaluate human-caused disturbances on a landscape scale has become a focus in the fields of geography, ecology and environmental science.

Early studies in the United States of America and England date back to the latter half of the 19th century when there were thriving tourism industries. Many qualitative studies have been conducted on aspects of tourism development and its influences on the vegetation, water, soil, air, architectural style, etc. of tourist destinations^18–21^. For instance, Wagar^22^ proposed an experimental simulation method based on a sample investigation and comparative analysis that simulated anthropogenic disturbances semi-quantitatively. Sun and Liddle^23^ further proposed the concept of tourism environmental impact and preliminarily and theoretically explored the ecosystem resisting capability and recovery mechanisms to human-related activities. As quantification methods are improved, some studies on tourism disturbances have evolved from a theoretical framework to analytical tools, with an emphasis on the applicable methods of landscape planning and management^24–27^. Studies have mainly focused on two aspects: analysing the cumulative negative effects of tourist destinations on regional environments through vegetation and soil changes^28–31^ and the semi-quantification of anthropogenic disturbance intensity by calculating several integrated indices, such as tourism environmental capacity, ecological environmental capacity, and ecological disturbance coefficients^32–36^.

Generally, traditional studies have mainly concentrated on the static and simple effects on one single aspect of a site or patch in tourist destinations, rarely considering the comprehensive impact of anthropogenic disturbances on landscape patterns and processes. In addition, methods of sample investigation or semi-quantitative landscape pattern metrics, which have relatively low data continuity and repeatability on the macro scale, are widely used. Other main limitations are that disturbance patterns change over time, and spatial pattern differentiation is frequently unavailable from previous research. In this study, we proposed an improved disturbance analysis method that involves temporal and spatial quantification factors. Within this framework, we constructed a landscape disturbance model to identify and quantify the main anthropogenic disturbances in typical tourist destinations. The index of the overall variation coefficient (*OVC*) is defined to evaluate the intensity of anthropogenic disturbances on a landscape scale, and the temporal trends of the differentiation of disturbance intensity in various tourism development phases is explored on a scale consistent with scenic spots. Then, the spatial differentiation characteristics of the disturbance intensity, as it relates to elevation, slope and spatial gross domestic product (GDP) are analysed to provide proactive countermeasures for greater resilience to regional tourism development.

## Study Area and Data Preparation

### Overview of the study area

The Li River Basin is located in Guilin, northeastern Guangxi Province, and flows in the north-south direction. The area of the basin is 5,306.06 km^2^, and the main branch, with a length of 164 km, flows through four counties (Xing’an, Lingchuan, Lingui and Yangshuo) and five regions (Yanshan, Diecai, Xiufeng, Qixing and Xiangshan). The basin has a mid-subtropical monsoon climate, with an average annual precipitation of 1,400~2,000 mm; the vegetation coverage rate of the region is approximately 62%, and the species are varied with notable vertical zonality. As the core area of regional, social and economic development, the Li River Basin has a high population density. At the end of 2015, it comprised over 60% of the total population of the city, and the regional GDP accounted for over 60% of the GDP in Guilin. The basin is also the centre of tourism in Guilin city, with 55 scenic spots above A level: 41 natural attractions and 14 human and cultural attractions. In 2015, the number of tourists was more than 30 million, and the total tourism income reached 41.60 billion RMB (Renminbi, Chinese yuan), accounting for 80% of the total tourism income in Guilin city. In general, the basin is a typical region that stimulates economic development through the development of tourism, which has been relying heavily on regional eco-environmental resources. In this way, exploring the interaction mode between tourist activities and the eco-environment is of significant importance for sustainable development.

### Data inputs

The land use/cover data, with a pixel resolution of 30 m × 30 m, comes from remote sensing images in three-periods (October 1989, September 2000, and October 2015) provided by a geospatial data cloud site of the Computer Network Information Centre, Chinese Academy of Sciences. Five types of landscapes, i.e., farmland, forest, grassland, water and construction land, are extracted using the supervised classification method in ENVI 4.7 (Exelis Visual Information Solutions, http://www.enviidl.com/). The landscape types are used as the basic data for the landscape pattern variation analysis. The DEM (digital elevation model) data, with a resolution of 30 m × 30 m, comes from the National Earth System Science Data Sharing Infrastructure. The sub-watersheds, elevation and slope are computed from the DEM. The basic data, such as roads, rivers, and residential areas with a map scale of 1:250,000, originate from the Geographic Information Database provided by the National Geomatics Centre of China. NPP-VIIRS data for 2015 were obtained from the Earth Observation Group, NOAA National Geophysical Data Centre^37^. The scenic spots data stems from *Guilin Lijiang Chorography*^38^, *Guilin Yearbook*^39^, the Guilin tourism information network and several thematic maps of tourism in the basin.

## Results and Analysis

### Landscape variation characteristics of different disturbance types

**Figure 1:**
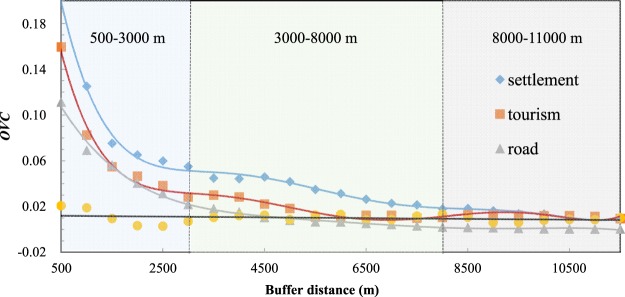
Results of the landscape structure variation analysis for different anthropogenic disturbance sources in 2015. The horizontal axis shows the value of the buffer distance in the landscape extraction model (Supplementary Note 1), and the vertical axis is the *OVC* value, which represents the intensity of the disturbances. The diamonds, squares and triangles are the *OVC* values of settlements, tourism and main roads, respectively, in a certain buffer distance, and the circles approaching the 0.01 value are the *OVC* values of the 40 random points in the natural background region. The corresponding lines are the fitting curves of the *OVC* for the four items estimated by a generalized polynomial model.

It has been generally acknowledged that anthropogenic disturbance can induce changes in landscape structure on a certain scale. Through the use of landscape disturbance models and spatial analysis tools, the landscape *OVC*, which represents the intensity of disturbances, has been plotted (Fig. 1). Compared with the undisturbed natural background line, which remains below 0.02, the *OVC* of the three anthropogenic disturbance types shows notable variation across a spatial gradient. Within a close distance (500~3,000 m), the values of tourism, residents and roads are relatively high, and the variation in *OVC* is substantial. When the buffer distance reaches a certain value (3,000~8,000 m), the *OVC* of the three anthropogenic disturbance types no longer decreases significantly and gradually approximates the value of the natural background region, showing that the landscape structure gradually changes to resemble the natural landscape. When the distance reaches approximately 11,000 m, the values of the three anthropogenic disturbance types become relatively stable and convergent, remaining similar to the background value (*OVC* ≤ 0.02). This indicates that beyond this distance, the buffer area is restricted by the natural background factors and that the anthropogenic disturbances can be ignored at this scale.

A comparison of the *OVC* among the three anthropogenic land cover types demonstrates the disparities in disturbance intensity. Of the variables examined, the maximum *OVC* of settlement is the largest (0.20), the maximum *OVC* of tourism is in the middle (0.16), and the *OVC* value of road is the smallest (0.11). The *OVC* line of road buffer becomes stable at an approximate distance of 3,000 m, and the tourism *OVC* line begins to narrowly fluctuate at a distance of 5,500 m, while the settlement disturbance becomes stable at approximately 7,500 m. This proves that both the maximum and average value of road *OVC* are far lower than those of the other anthropogenic disturbance types, while the disturbance of the local settlements on the surrounding landscape has a relatively strong penetrative and diffused ability. The influential sphere of tourism disturbance is also relatively large, indicating that the disturbances created by tourism activities that centre on the scenic spots are influential throughout the basin. The spatial distribution of the three types of anthropogenic disturbances are described in Supplementary Note 2.

### Temporal variation in disturbance intensity in different periods of regional development

**Figure 2:**
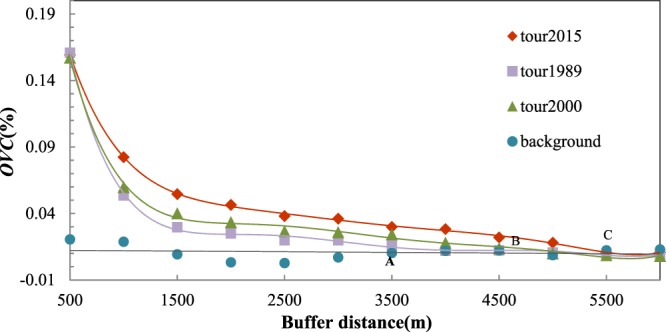
Variation in tourism disturbance intensity in different periods. The diamonds, triangles and squares are the *OVC* values of tourism disturbances in 1989, 2000 and 2015, respectively, and the circles approaching the 0.01 value are *OVC* values of the 40 random points in the natural background region. The corresponding lines are the fitting curves of the *OVC* for the four items estimated by a generalized polynomial model. A, B, and C are the breakpoints of the tourism *OVC* approaching the natural background value.

The landscape disturbance model showed differences as well as similarities in the comparison of the variation in the overall and classified landscape patterns in 1989, 2000 and 2015. Figure 2 shows that the maximum tourism *OVC* during the three years was almost the same (0.16), meaning that the maximum disturbance intensity in local regions during the 26 years remained relatively stable. The variation in the three *OVC* lines differs: in 1989, the tourism disturbance line becomes stable at point A (an approximate distance of 3,500 m); in 2000, the tourism line begins to narrowly fluctuate at point B (a distance of 4,500 m); and in 2015, the tourism line approaches the background value at a distance of 5,500 m, which means that the influential sphere of tourism disturbance has been spreading over time. As an increasing number of scenic spots were constructed or expanded during the 26 years, tourism disturbance in the region displayed evident variation and fluctuation.

**Figure 3:**
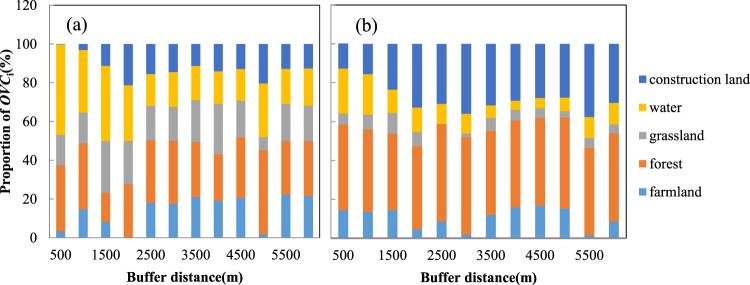
Differentiation of the effects of tourism disturbance intensity on landscape patterns in the periods of 1989–2000 (**a**) and 2000–2015 (**b**). A bar filled with a given colour is the proportion of *OVC*_*i*_ of each landscape type in a fixed buffer distance, which refers to the disturbance intensity in a certain landscape type.

To further explore the variation in the landscape patterns in two periods, the composition of landscape *OVC* is calculated. Specifically, the differentiation of *OVC*_*i*_ of the five landscape types in the 1989~2000 and 2000~2015 periods was plotted (Fig. 3) and displayed broad consistency with the phasic tourism development in the basin. According to the statistical data and spatial layout information collected from *Guilin Yearbook* and the local Tourist Administration, 11 scenic spots were newly built or renovated during the period of 1989 to 2000 (shown in Fig. S3 in Supplementary Note 3). Most of these were natural scenic areas located along the shore of the Li River. For example, the construction of the Gudong Waterfall and the transformation of the Julong Lake scenic area, whose curves show considerable peak values in Fig. 3(a), caused relatively drastic disturbances on the water nearby as well as the surrounding forest and grassland. After 2000, there were 27 newly built scenic spots and 29 spots were transformed in the last 15 years, such as the Folk Cultural Garden, the Dayu Ancient Town and Butterfly Spring (as shown in Fig. S3 in Supplementary Note 3). These were mainly distributed in the downtown area of Guilin and Yangshuo County. The disturbance sphere in this period is much larger than that in the former period, the disturbed landscape type is not restricted to forestland, and the proportion of construction land shows a constantly high value with greater fluctuation. This is not only subject to the spatial concentration of scenic area construction in the urban area, but is also directly related to the prosperity of cultural attractions, such as resort villas, cultural sites, and theme parks.

### Spatial differentiation characteristics of anthropogenic disturbances

**Figure 4:**
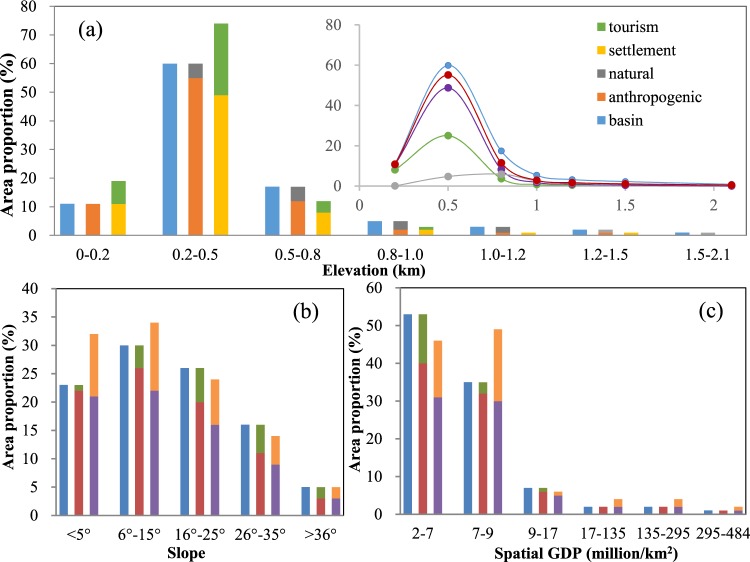
Spatial differentiation of tourism disturbances, settlement disturbances, natural background areas, anthropogenic disturbances and the whole basin, relative to the factors of elevation, slope and spatial GDP. The horizontal axis shows the classified value of each spatial factor, and the vertical axis indicates the proportion of the area of a certain type (including areas disturbed by tourism, settlement, anthropogenic factors, natural background area, and the whole basin) in a fixed range and the whole basin. The inset in (**a**) shows the variation in the five items along with elevation corresponding to (**a**).

The spatial distribution of the disturbed areas in each land cover type showed stratified heterogeneity consistent with the three disturbance factors in the basin. As shown in Fig. 4(a), the distribution of anthropogenically disturbed areas by elevation displays a phenomenon of polarization: the disturbed areas located at 200–500 m elevation, which is the most intensive area of settlement and tourism disturbances in the basin, account for over 50% of the whole basin. The disturbed areas located above 1,000 m occupy less than 5% of the basin, meaning that over a certain elevation, the anthropogenic disturbance is minimal and the region is mainly controlled by natural background factors. The inset in Fig. 4(a) displays the variation in the proportion of the areas of the five land cover types to further reveal the relationship between the distribution of anthropogenic disturbance and that of the whole basin. All of the disturbance types have curves that approximate a normal distribution in the low elevation regions and slowly varying curves that approximate the vertical axis at high elevations.

The variation in the proportions of the area of the five land cover types related to slope is lower than that related to elevation and GDP. As Fig. 4(b) shows, the areas with anthropogenic disturbances are distributed in almost all the slope ranges, and the variation resembles that of the whole basin. This indicates the generality and diversity of human activities in the basin, which show a slight increase at first, followed by a remarkable decrease. The areas with the most intensive anthropogenic disturbances have a slope ranging from 6°–15°, while 70% of the whole basin has a slope below 35°. This suggests that residential and tourism construction activities mainly occur in the relatively flat areas. A comparison of the settlement-disturbed area with the tourism-disturbed area found the same variation as was observed for the change in slope. The addition of the two types of disturbance would lead to a higher value than that obtained for the absolute area of anthropogenic disturbance in most ranges. This indicates that the two types of disturbances might have superimposed effects in some areas, further adding to the complexity of anthropogenic disturbances.

The distribution of anthropogenically disturbed areas related to spatial GDP has shown typical characteristics of step-wise development. As displayed in Fig. 4(c), the proportion of low GDP areas accounts for more than 80% of the entire basin, and the variations in the proportion of disturbed area in corresponding regions are not that extensive. When the GDP value reaches 9 million/km^2^, the absolute area with anthropogenic disturbances decreases sharply, which is consistent with the GDP distribution of the entire basin. When the GDP value is greater than 17 million/km^2^, which is the average GDP value of the basin, the variation in the disturbed area returns to a minimal state; however, the absolute proportions of areas with anthropogenic disturbances reach approximate 100% of the overall area of the basin. This means that the regions with higher socio-economic conditions have been easily disturbed by human activities. These phenomena are tightly related to the spatial patterns of tourism development in the basin. There is a polarization in the development of the social and economic conditions in the basin, and areas with relatively low levels of economic development, such as Xing’an and Linchuan counties in the northern part of the region, have lagged in tourism development. In these areas, tourism attractions that are mostly dependent on local traditional resources, such as the Dongjiang Forest Resort Villa and the Dayeshenjing Ecological Tourism Scenic Area, which are easily disturbed by human activities due to their fragile ecosystems, are found. Areas with higher economic intensity, represented by downtown Guilin and Yangshuo, have abundant tourism attractions as well as a relatively large population, which leads to a severely disturbed landscape.

## Discussion

### Landscape variation characteristics of different disturbance types

Anthropogenic disturbances in the basin were effectively quantified and distinguished from natural disturbances by the use of a landscape disturbance model and the *OVC* index. Compared with natural background disturbance, anthropogenic disturbances have a higher intensity and notable variation in the regional landscape structure. The analysis of the sphere of influence of the three typical disturbance sources shows that tourism and settlements have been the main anthropogenic disturbances affecting the landscape structure. The intensity of disturbance from settlements in the basin is the highest, which indicates that traditional, continuous and complicated production and living activities, such as cultivation, the felling of trees and infrastructure construction, have had a relatively strong, penetrative impact on landscape structure variation. The intensity and influential sphere of disturbance driven by tourism has approached those of the disturbance driven by settlements, indicating that the rapid tourism development in recent decades, which centres on the construction of scenic spots, is becoming the main type of anthropogenic disturbance in the basin. The diffusion effects of tourism disturbance have necessitated new requirements for the configuration of scenic spots.

### Temporal characteristics of changes in disturbance in different periods of tourism development

Further analysis of the variation in disturbances driven by tourism in different periods has shown broad consistency with phasic tourism development in the Li River Basin. A review of the socio-economic development and management policy of Guilin City showed that the year 2000 is a crucial breakpoint for tourism development in the region. In 2000, the *Overall Planning of Tourism Development in Guilin* was issued, and a series of institutional framework innovation policies, such as tourist agency marketization and the standardization of scenic spot construction, operation and management, were adopted^40^. After 2000, up to 56 scenic spots were built or transformed, and billions of RMB were invested to develop infrastructure and enrich tourism products. Apart from the proliferation of scenic spots, the development of tourism affects the patterns of landscape disturbance to a great extent: the construction of natural resource-based attractions in the region causes strong disturbances of aquatic areas, forestland and farmland, while the development of artificially constructed scenic spots tends to influence construction land more significantly. This indicates that regional tourism development faces trade-offs between the protection of ecological land and the expansion of construction land^41^. With basin tourism approaching a holistic development stage, the effects on the landscapes upon which tourism relies will continue to increase.

### Spatial differentiation characteristics of anthropogenic disturbances

In general, the disturbance pattern was restricted by local natural factors and socio-economic conditions, while some parts of the area showed specific changes. This phenomenon conforms to the principles of landscape ecology^42^. The curves of the proportions of disturbed area related to the three spatial factors all displayed points of inflection, and the proportion decreased sharply above a threshold value. Tourism disturbances occur in almost any natural range because the scenic spots are widely distributed in the basin, mainly in areas with a low elevation and gentle slope, where the residents’ daily activities are also concentrated. In these regions, the two types of disturbances have a compounding effect, thus further increasing the complexity of the effect of disturbances on regional landscape. Furthermore, in areas with less developed natural environments, the intensity of anthropogenic disturbances could not be neglected. Since karst landforms develop well in the Li River Basin, which has a steep slope, thin soil and low vegetation coverage, tourism and settlement disturbances seriously threaten ecosystem stability, leading to soil erosion and habitat destruction, especially in primitive areas with a fragile ecosystem and unsound countermeasures.

### Tourism management implications

Considering the temporal and spatial characteristics of anthropogenic disturbances in the basin, two typical patterns of tourism development were identified. The first pattern is found in northern Xing’an. With the characteristics of relatively high elevation (over 1,000 m) and steep slope (mostly above 25°), the eco-environmental sensitivity of this region is high, and the economy is underdeveloped, so tourism development is highly dependent on natural resources^41^. As the trend of “eco-tourism” has increased in recent years, these relatively remote areas have gradually become the hotspot of basin tourism, and the disturbance intensity has been increasing and threatening the local natural ecosystems. Therefore, strict protection and management measures should be carried out accordingly. With tourism development gradually entering the stage of long-term planning in these regions, the optimization of tourism products and structures, i.e., the transition from the construction of single scenic spot to tourist industry brand development and the conversion from a high dependence on natural resources to the deep mining of folk culture, is an effective measure to relieve landscape disturbances in the basin.

Another development pattern is represented by the downtown areas of Guilin and Yangshuo. These areas have a relatively sound natural environment, developed economy, and abundant tourism products. Large-scale scenic spots construction and traditional residential activities are the main sources of disturbances, exerting considerable impacts on the variation in the regional landscape structure and pattern. The superimposed effects of the two disturbance sources in some areas have further added to the disturbance complexity. As the tourism industries in these counties continued to grow and more activities that exploit resources were established, the influential sphere of tourism disturbance spread. For instance, in some areas with intensive tourism resource use, the distance between scenic spotss is little more than 1,000 m, far less than the steady-state sphere of disturbance driven by tourism (3,500 m-5,500 m). This undoubtedly causes interference and damage to the surrounding landscape pattern. Therefore, in addition to greatly restricting scenic spots construction and management, exploring the coordination mechanism between tourism development and local residential life in the basin and fully considering the rationality of scenic attraction configuration are important considerations to relieve anthropogenic disturbances.

### About the spatial scale

A review of previous studies on anthropogenic disturbances in tourism destinations verified the validity of the results. For instance, Arocena *et al*. reported visitor-induced changes to the chemical composition of soils in Mt Robson Provincial Park (Canada), which can be detrimental to soil quality and the overall health of the ecosystem^28^. Kissling *et al*. found that tourist trampling reduced the species density and altered the composition of soil nutrients, and thus ultimately led to less available nutrients for plant uptake^29^. Li *et al*. proved that anthropogenic disturbances and excessive tourism development have affected soil infiltration, which was related to the degradation and fragmentation of a fragile meadow on Wugong Mountain^31^. However, these studies mainly concentrated upon the simple effects on one single aspect of the environment in tourist destinations, with methods based either on sample investigation or on very small patch scales, the findings of which are difficult to extrapolate to macro scales. As a result, the comprehensive impact of anthropogenic disturbances on landscape patterns and processes could hardly be demonstrated directly. The approach in this study adopts the landscape scale and a unique disturbance model to identify and quantify the main anthropogenic disturbances in tourist destinations, evaluates the intensity of different disturbances and then detects the temporal and spatial variation characteristics on a scale consistent with scenic spotss. This approach explicitly displayed the heterogeneity of anthropogenic disturbances on landscape patterns and provided a better understanding on sound spatial scales, which could provide information for the development of regional tourism management countermeasures.

## Conclusion

This research explored the identification and quantification of anthropogenic disturbances in tourist destinations through the construction of a landscape disturbance model. The variations in the disturbance intensity with the spatial and temporal factors were assessed using the *OVC* index. The results of the model are consistent with the phasic development of the social economy in the Li River Basin in general, and further comparison and classification analysis has revealed the remarkable influences of anthropogenic disturbances on landscape patterns. Specifically, settlements and tourism have been the significant anthropogenic disturbances in the basin, and the general tendency of the variation in the disturbance intensity is restricted by local natural and socio-economic conditions. The characteristics of the different landscape types are closely related to the tourism development pattern. For instance, the distribution of natural resource-based and artificially constructed scenic attractions, the superposition disturbances of settlements and tourism, and the degree of dependence of tourism activities on local natural resources are all vital factors that influence the disturbance intensity and its variation. With basin tourism entering a holistic development stage in recent years, the disturbance effects in the landscapes upon which tourism relies will continue to increase; thus, a sound trade-off between the protection of ecological land and the expansion of construction land is urgently needed^41^. In addition to the restriction of extensive tourism construction, future tourism management should explore optimizing tourism development patterns and enriching tourism cultural products to achieve the sustainable management of watershed tourism.

Future studies should focus on landscape morphological changes under anthropogenic disturbances, which could further demonstrate the dynamic process of disturbance. Additionally, the landscape in the basin is classified into five types in this study, owing to the complicated topography and relatively low-resolution land use images, which limits the detailed pattern analysis to some extent. Higher resolution data and improved classification methods could be employed in future studies to improve landscape classification accuracy and, subsequently, the model results.

## Materials and Methods

### Extraction of landscape types

To obtain land use/cover data for the basin in the three years examined, a supervised classification was performed in the ENVI 4.7 platform (Exelis Visual Information Solutions, http://www.enviidl.com/). First, regions of interest that could represent different spectral types from the landscape, which serve as the training areas for the classification model, are selected with the ROI tool. Then, plots and statistics of the training areas, such as histograms and ROI separability, are constructed and calculated for each class and are used to evaluate and modify the availability of the training samples. From these samples, the signature file of each landscape type is created. After that, a neural network algorithm is used to obtain the classification results, and the process is repeated and improved by editing the signature files and rerunning the classification model until the overall accuracy meets the needs of the research. Eventually, five landscape types, i.e., farmland, forest, grassland, water and construction land, are extracted and used as the basic data for the landscape pattern variation analysis.

### Identification of disturbance sources and buffer areas

**Figure 5:**
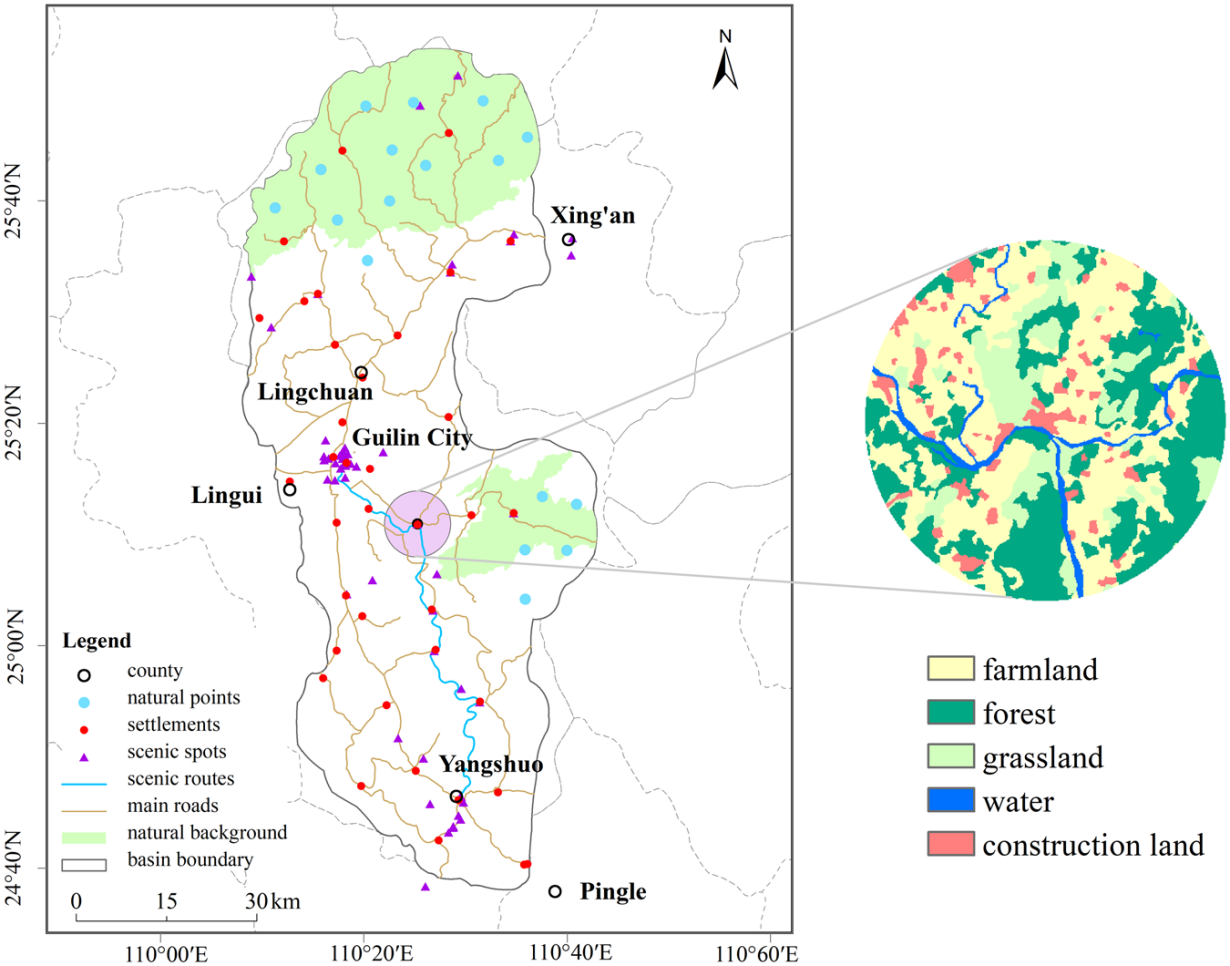
Distribution of disturbance sources in the Li River Basin. The red circle on the map represents settlement disturbance sources, the violet triangle represents scenic spots disturbance sources, the solid sky blue line represents scenic route disturbance sources, the solid light sienna line represents road disturbance sources, and the apatite blue circle represents natural background points. The right circle in the inset is a local magnification of the landscape structure of an area with a settlement disturbance at a distance of 5,500 m, where five different colours represent the five landscape types. The basin boundary in the map is identified according to the *Li River Basin Ecological Environment Protection Regulations in Guangxi Autonomous Regions*, and the border of the Haiyangshan Water Conservation Forest is determined by delineating sub-watersheds based on DEM data. This map was generated using ArcGIS 10.2 software, http://www.esri.com/.

Landscape evolution is restricted by two types of heterogeneous factors: natural conditions and human activities^43^. The influence of these two factors on the landscape has additive properties, i.e., natural conditions are fundamental in landscape structure changes, while human activities affect landscape patterns directly and purposefully with higher intensity^42^. Considering the regional developmental features of the Li River Basin, tourism has become an important and overwhelming industry in the basin, thus exerting considerable anthropogenic disturbances on the regional eco-environmental resources. In addition, traditional production and residential activities have continuous and complicated influences on the variation in landscape structure over time. Additionally, road disturbances cannot be neglected in the mountainous watershed, because roads are crucial landscape corridors, near which the majority of human activities congregate. Therefore, three possible types of anthropogenic disturbances are proposed: (a) linear disturbance spreading from the main road towards both sides; (b) punctuated disturbance spreading from the settlements towards the surroundings; and (3) linear and punctuated disturbances spreading from the important scenic spotss and routes to the surroundings. The Maoershan Natural Reserve, located in the northern part of the basin, and the Haiyangshan Water Conservation Forest east of the downtown area are chosen as the natural background regions for a contrast analysis, as the two areas are little affected by human activities. Specifically, roads of county and town scale are selected as road disturbance sources; township and county locations are chosen as the settlement disturbance sources; and 56 scenic spotss whose grades are A and above are selected as tourism disturbance sources. Moreover, 40 random points in the two natural regions are selected as the background locations for the contrast analysis. The spatial distribution of disturbance sources and the results of the buffer process in the Li River Basin are displayed in Fig. 5.

### Analysis of the disturbance intensity based on the landscape disturbance model

According to Zeng’s research^44^, anthropogenic disturbances have a tendency to spatially concentrate around a particular landscape, and the main influential sphere of the disturbance is the buffer area centred on the disturbance sources, in which the landscape structure differs remarkably from that of the surrounding area. Beyond the influential sphere of anthropogenic disturbances, the landscape is mainly affected by natural factors, and the structural variation is not notable. In this study, landscape variation coefficients were adopted to quantify the effect of various anthropogenic disturbances on landscape structure. This effect was measured through the index of the overall variation coefficient, calculated as follows:

1 $$OVC={\sum }_{i=1}^{n}|{a}_{i}-{a}_{i}^{^{\prime} }|$$

where *OVC* is the overall variation coefficient of all the landscape types, *i* represents landscape type, and *a*_*i*_ and *a*’_*i*_ are the proportional areas of landscape *i* in two adjacent buffers.

After calculating the *OVC* value for different buffer distances, the distance attenuation curves of certain anthropogenic disturbance intensities could be simulated. Generally, in short-distance buffers that are distributed near the disturbance source, the landscape is strongly disturbed, and the value of *OVC* always fluctuates drastically. When the buffer distance reaches a certain value, the intensity of anthropogenic disturbances decreases markedly, while the value of *OVC* fluctuates narrowly around the background value without obvious decline, indicating that the landscape structure beyond this distance resembles natural background conditions. Among the buffers with the same conditions, the higher the *OVC* value, the more notable the landscape structure variation^45^.

In this study, the initial radius of each buffer is set at 500 m to effectively reflect the trends of landscape structure change, and 20, 30, and 40 buffers are generated by adding 500 m successively. The change in landscape structure tends to be stable beyond a distance of 12,000 m, i.e., the 24^th^ buffer region. Therefore, we set 12,000 m as the maximum buffer distance, extracted the landscape information in the range of each buffer and computed the variation coefficients of adjacent buffers using the modelling and scripting language tools in ArcGIS 10.2 software (Environmental Systems Research Institute, Inc, http://www.esri.com/). This process is described in detail in Supplementary Note 1.

### Temporal variation analysis of tourism disturbance

The value of the landscape *OVC* in different periods can reflect the temporal variation in a certain disturbance type, while the composition of the landscape *OVC* in a certain period can represent the variation in intensity of each landscape type: the higher the value, the stronger the disturbance intensity of the landscape type. The equation is as follows:

2 $$OV{C}_{i}=\frac{|{a}_{i}-{a}_{i}^{^{\prime} }|}{{\sum }_{j=1}^{n}|{a}_{j}-{a}_{j}^{^{\prime} }|}$$

where *OVC*_*i*_ is a proportion of the variation coefficient of landscape *i*, *a*_*i*_ and ${a}_{i}^{^{\prime} }$ are the area proportions of landscape *i* in two adjacent buffers, and *a*_*j*_ and ${a}_{j}^{^{\prime} }$ are the area proportions of landscape *j* in two adjacent buffers.

To consider the characteristics of tourism development in different phases, 1989, 2000 and 2015 are selected for the overall and classified temporal variation analysis in the basin. The newly built scenic spotss within the periods of 1989–2000 and 2000–2015 are used as the added disturbance sources, and the differentiation of the *OVC*_*i*_ value of five landscape types in the two phases was computed. Specifically, we set 300 m and 6,000 m as the initial buffer distance and the maximum buffer distance, respectively, to reflect the subtle variation in landscape structure.

### Spatial variation analysis of tourism disturbance

The composition of regional disturbed areas related to spatial factors could reflect the spatial differentiation of a specific disturbance type. Considering the high elevation differences and dramatic topographic changes in the basin, elevation and slope are selected as the regional natural environmental indicators to show the natural background condition in the basin^46^, while spatial GDP, which is directly correlated with the economic level and population distribution in the tourism destination area^47^, is chosen as the indicator of socio-economic variation. In view of the macroscopic trend analysis, the slope is classified into 5 ranks referring to the *general principles of the soil and water conservation plan*^48^. The elevation is reclassified into 7 ranks according to the national geo-information survey guideline in China, and GDP data are reclassified into 6 ranks by comparison with the level of socio-economic development within a nearby county. The specific data are shown in Table 1. Using the spatial quantification and analysis methods, we obtained the spatial differentiation of the two anthropogenic disturbance types related to elevation, slope and spatial GDP in the basin. The method and analysis are described in detail in Supplementary Note 4.

**Table 1:** The classification of slope, elevation and spatial GDP in the basin.

Spatial factor	Value range	Classification results
Slope	0–75°	<5°
		6°–15°
		16°–25°
		26°–35°
		>36°
Elevation	0–2,117 m	0–200 m
		200–500 m
		500–800 m
		800–1,000 m
		1,000–1,200 m
		1,200–1,500 m
		1,500–2,117 m
Spatial GDP	214–48,413 million/km^2^	214–647 million/km^2^
		647–926 million/km^2^
		926–1684 million/km^2^
		1,684–13,499 million/km^2^
		13,499–29,489 million/km^2^
		29,489–48,413 million/km^2^

## Supplementary information

supplementary material

## Data Availability

The datasets generated during the current study are available from the corresponding author on reasonable request.
