# Supplementary material for: Spatio-temporal Analysis of Anthropogenic Disturbances on Landscape Pattern of Tourist Destinations: a case study in the Li River Basin, China
Source: Sci Rep. 2019 Dec 17;9:19285. doi: 10.1038/s41598-019-55532-w (PMC6917706; doi:10.1038/s41598-019-55532-w)
Supplement: Supplementary file 1 — supplementary material [file 41598_2019_55532_MOESM1_ESM.pdf]

Supplementary Information

Spatio-temporal Analysis of Anthropogenic Disturbances on Landscape Pattern of Tourist Destinations: a case study in the Li River Basin, China

Yunyun Xiang[1,*], Jijun Meng[2], Nanshan You[3], Peixiong Chen[1] & Hui Yang[1]

[1]*Second Institute of Oceanography, Ministry of Natural Resources, Hangzhou 310012,China.*

[2]*School of Urban and Environmental Sciences, Key Laboratory for Earth Surface Processes, Ministry of Education, Peking University, Beijing 100871, China.*

[3]*Institute of Geographic Sciences and Natural Resources Research, CAS, Beijing 100871, China.*

**Corresponding author: xiangyunyun@126.com*

Supplementary Note 1: Process of extracting landscape pattern information from each buffer.

The model of extracting landscape type information from each buffer under a certain disturbance source lists in Fig. S1.

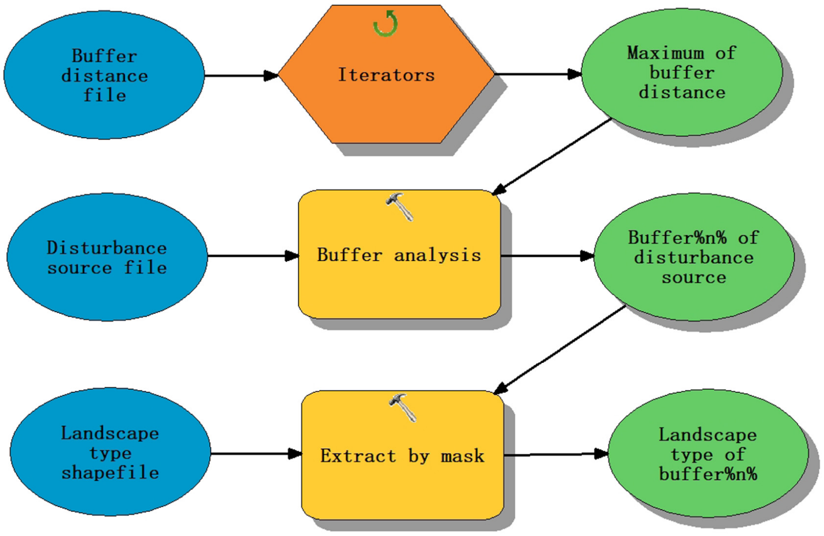

Fig. S1 Model of extracting landscape type information from each buffer under a certain disturbance source. The buffer distance file is the dataset of buffer distance from the initial radius of each buffer to the maximum. Iterators control the iteration of the whole model by adding a fixed interval of buffer distance successively until the distance (represented by variable n) reaches the maximum. The disturbance source file is the linear or punctate or the multiple dataset of a certain disturbance source type, and landscape type shapefile represents the distribution of the five landscape type in one year. Through the “Buffer analysis” tool, the buffer polygons around input features to a

specified distance (represented by variable buffer%n%) are calculated. And the tool of "Extract by mask" helps to get the landscape structure of each buffer. This figure was generated using ArcGIS 10.2 Model Builder module, http://www.esri.com/.

Firstly, considering the divergences in different types of anthropogenic disturbance sources, the algorithm of "Buffer analysis" for the three main types are determined: road disturbing process is represented by linear buffer spreading from the main road towards both sides; settlements disturbing process is measured by circular buffer spreading from the punctate settlements towards surroundings; tourism disturbing process is simulated by the combination of linear and circular buffer spreading from the important routes and scenic spots to surroundings. Secondly, for the preoperation of the model, the initial radius of each buffer is set at 200 m, 300 m, 400 m, and 500 m successively, and the maximum buffer distance is set at 20,000 m, to explore the trends of landscape structure change. Then the landscape structure of each buffer in consecutive distances under the three types of anthropogenic disturbance sources is acquired by running the model, and the *OVC* value in different buffer distances is calculated by formula (1) in the manuscript using Scripting Language and VBA tools. After that, the distance attenuation curves of certain anthropogenic disturbances intensity are plotted, and it turns out that the change of landscape structure in 2015 tends to be stable beyond the distance of 12,000 m, and the interval of 500 m could effectively reflect the trends of landscape structure change. Therefore, we set 500 m and 12,000 m as the initial radius and maximum buffer distance respectively, and 24 buffers are generated by adding 500 m successively. In this way, the landscape variation coefficients of adjacent buffer under the three types of disturbance sources are computed.

Supplementary Note 2: the spatial distribution of anthropogenic disturbances.

The spatial distribution of the three types of anthropogenic disturbances lists in Fig. S2.

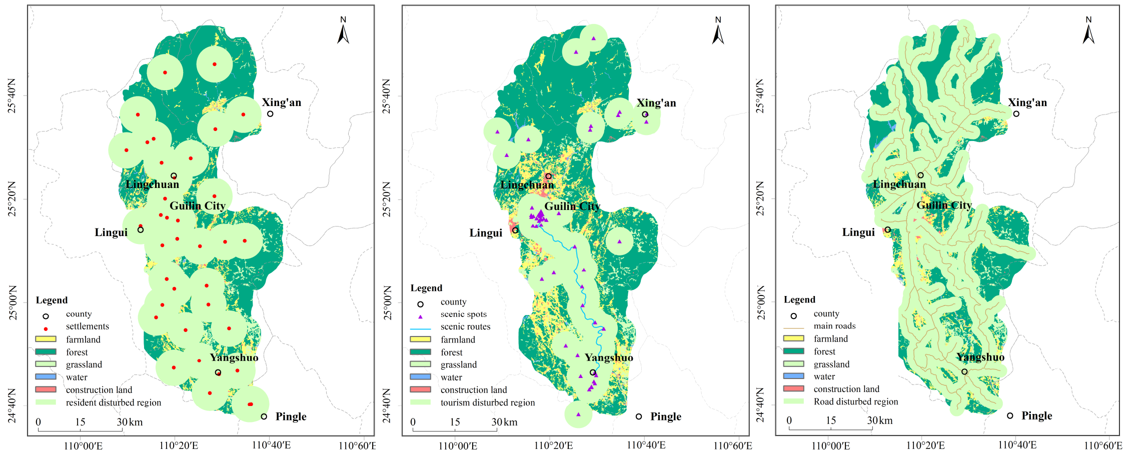

Fig. S2 Distribution of residents, tourism and roads disturbance in Li River Basin. The three maps display the disturbed areas under the sources of residents, tourism and roads respectively, which are displayed by light green polygon. Road disturbance shows linear structures spreading from the main road towards both sides; Residents and tourism disturbances show the characteristics of extending from the spots and routes towards surroundings. This map was generated using ArcGIS 10.2 software, http://www.esri.com/.

Supplementary Note 3: the spatial distribution of scenic spots in different periods.

The spatial distribution of the scenic spots and the tourism influential sphere in the year of 1989, 2000 and 2015 list in Fig. S3.

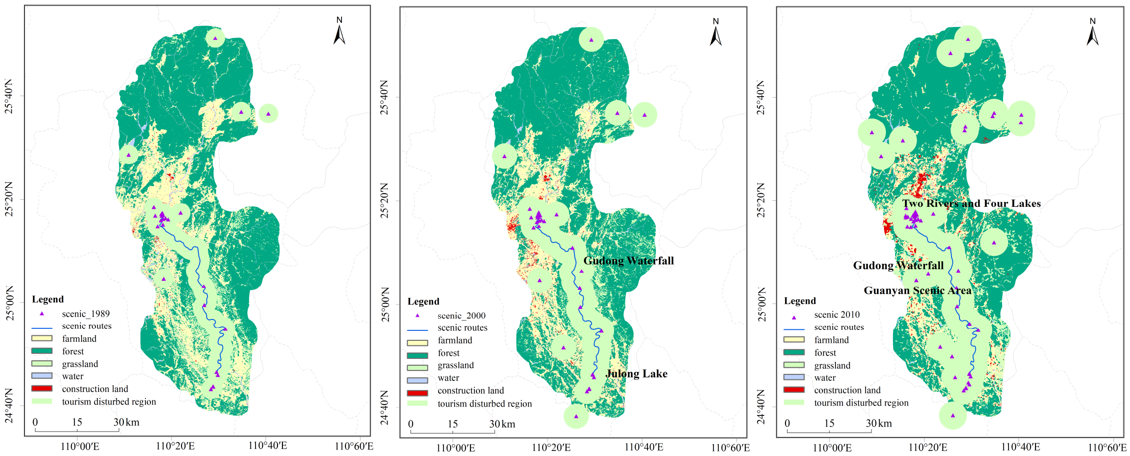

Fig. S3 Distribution of the scenic spots and tourism influential sphere in the year of 1989, 2000 and 2015. The three maps display the scenic spots and disturbed areas under the sources of tourism respectively, which are displayed by light green polygon. The tourism influential sphere is approximate to 3,500 m, 4,500 m and 5,500 m in the three years. This map was generated using ArcGIS 10.2 software, http://www.esri.com/.

Supplementary Note 4: the quantification and analysis of disturbances variation with three spatial factors.

The elevation and slope information of the basin is computed in the platform of ArcGIS10.2 using the "Surface analyst" tool based on the DEM data of the basin. To reflect intra-regional economic distribution, we introduce the Light model and the concept of "spatial GDP" in calculating the density of GDP in the basin. Specifically, we relate the output of agriculture, forest and husbandry to the landscape types of farmland, forest and grassland in the area and the output of secondary and tertiary industries to the construction land, to get the GDP value of each landscape type in every county.

The formula for the GDP value of farmland, forest and grassland is as follows.

$$G_{ik} = G_j / Count(i) \qquad (1)$$

Where, G_{ik} is the GDP value of land type *i*（*i=1~3*, represents the three kinds of landscape）in county *k*（*k=1~9*, represents the four counties and five regions in the basin）, G_j（*j=1~3*）is the total production of the primary industry in the county, $Count(i)$ is the grids total of landscape *i*。

In view of the remarkable interdependency between the nighttime light images and social-economy factors（Ghosh et al, 2010）, the light intensity factor is used to revise the GDP value of the construction land. And the following simplified linear model is used to allocate the value for the sample size is relatively small:

$$\rho_{ik} = DN_{ik} / \sum_{i=1}^{n} DN_{ik} \qquad (2)$$

$$G_{ik} = \rho_{ik} * G_k \qquad (3)$$

Where, ρ_{ik} is the proportion of light intensity of construction grid *i* in county *k*（*k=1~9*）, DN_{ik} is the DN（Digital Number）value of construction grid *i* in the county, G_{ik} is the GDP value of construction grid *i*, and G_k is the total production of the second and tertiary industry in the county.

After getting the absolute value of spatial GDP, the level of socio-economic development within the county and other similar areas of the basin are used to grade the value. And the spatial distribution of elevation, slope and spatial GDP shows in Fig. S4.

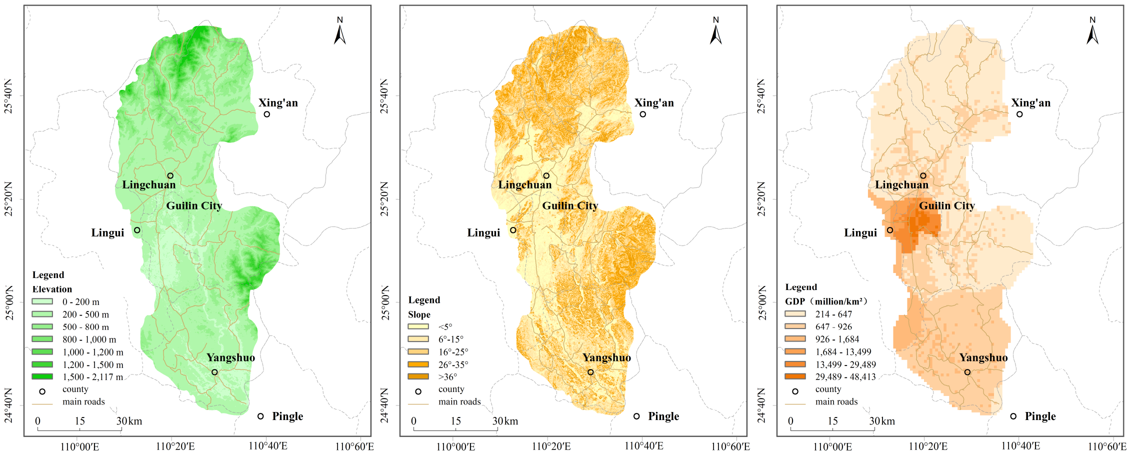

Fig. S4 Distribution of elevation, slope and spatial GDP in the Li River Basin. The three maps display the spatial differentiation of elevation, slope and spatial GDP respectively, with deep color representing high value and the light shade representing the low. This map was generated using ArcGIS 10.2 software, http://www.esri.com/.

Then we overlay the three maps with the area disturbed by tourism and settlement activities (displayed in Fig. S2), and extract the spatial differentiation information of tourism disturbances, settlement disturbances and natural background areas with the three factors by calculating the area proportion of five statistics (including tourism disturbed area, settlement disturbed area, natural background area, anthropogenic disturbed area and the whole basin), using the "Zonal Spatial Statistics" tool in ArcGIS 10.2 software, http://www.esri.com/. The results are plotted in Fig.4.

References

[1] Ghosh T, Powell R, Elvidge C D, et al. Shedding Light on the Global Distribution of Economic Activity[J]. The Open Geography Journal, 2010, (3):148-161.
